# Genetics of Inherited Retinal Diseases in Understudied Ethnic Groups in Italian Hospitals

**DOI:** 10.3389/fgene.2022.914345

**Published:** 2022-06-28

**Authors:** Paolo Enrico Maltese, Leonardo Colombo, Salvatore Martella, Luca Rossetti, Said El Shamieh, Lorenzo Sinibaldi, Chiara Passarelli, Andrea Maria Coppè, Luca Buzzonetti, Benedetto Falsini, Pietro Chiurazzi, Giorgio Placidi, Benedetta Tanzi, Matteo Bertelli, Giancarlo Iarossi

**Affiliations:** ^1^ Magi’s Lab S.R.L., Rovereto, Italy; ^2^ Department of Ophthalmology, ASST Santi Paolo e Carlo Hospital, University of Milan, Milan, Italy; ^3^ Department of Medical Laboratory Technology, Faculty of Health Sciences, Beirut Arab University, Beirut, Lebanon; ^4^ Translational Cytogenomics Research Unit, Bambino Gesù Children’s Hospital, IRCCS, Rome, Italy; ^5^ Rare Disease and Medical Genetics, Bambino Gesù Children’s Hospital, IRCCS, Rome, Italy; ^6^ Department of Ophthalmology, Bambino Gesù Children’s Hospital, Rome, Italy; ^7^ Fondazione Policlinico Universitario “A. Gemelli” IRCCS/Universita’ Cattolica del S. Cuore, Ophthalmology Unit, Rome, Italy; ^8^ UOC Genetica Medica, Fondazione Policlinico Universitario “A. Gemelli” IRCCS & Istituto di Medicina Genomica, Universita’ Cattolica del S. Cuore, Rome, Italy; ^9^ MAGI Euregio s.c.s., Bolzano, Italy

**Keywords:** inherited retinal diseases, understudied ethnic groups, genetic epidemiology, next generation sequencing, sanger sequencing

## Abstract

**Purpose:** Describing the clinical and genetic features of an ethnically heterogeneous group of (inherited retinal diseases) IRD patients from different underrepresented countries, referring to specialized Italian Hospitals, and expanding the epidemiological spectrum of the IRD in understudied populations.

**Methods:** The patients’ phenotypes underwent were characterized by exhaustive ophthalmological examinations, including morpho-functional testing. Genetic testing was performed using next-generation sequencing (NGS) and gene sequencing panels targeting a specific set of genes, Sanger sequencing and—when necessary—multiplex ligation-dependent probe amplification (MLPA) to better identify the genotype. When possible, segregation analysis was performed in order to confirm unsolved cases.

**Results:** The article reports the results of the phenotypes and genotypes of 123 IRD probands, 69 males and 54 females, mean age 41 (IQR, 54–30) years, disease onset at 13 (IQR, 27.25–5) years. Thirty-three patients out of 123 (26.8%) were Africans (North/Northwest Africa), 21 (17.1%) Asians, 19 (15.4%) Americans (South/Central America) and 50 (40.7%) Europeans (Eastern Europe). Retinitis pigmentosa was the most represented phenotype (56%), followed by cone dystrophy (11%) and Leber congenital amaurosis (7%), while *ABCA4* was the most frequently mutated gene (18%), followed by *USH2A* (9%) and *RPGR* (5%). About *ABCA4* variants found in Stargardt disease, macular and cone dystrophies were predominant in Asian (42%) and European (21%) patients. The most represented inheritance pattern was autosomal recessive, while a higher frequency of homozygous patients versus compound heterozygotes as compared to previous studies on Italian IRD patients was evidenced, reflecting a possible higher frequency of inbreeding marriages.

**Conclusion:** Though limited by the relatively low number of patients, the present paper paints a picture of the clinical and genetic features of IRD patients from understudied ethnic groups referred to Italian specialized hospitals and extended the epidemiological studies on underrepresented world regional areas.

## Introduction

Inherited retinal diseases (IRDs) represent a heterogeneous group of disorders, characterized by different retinal cells primarily affected, age of onset, disease progression, and mode of inheritance, with a syndromic or non-syndromic clinical presentation. Depending on the retinal cells primarily involved in the degenerative process, they are classified in retinitis pigmentosa (RP, also known as rod-cone), cone-rod, and macular dystrophies. Most of these forms are degenerative, but some phenotypes, such as congenital stationary night blindness (Zeitz et al., 2015) and achromatopsia (Tsang and Sharma, 2018), are generally considered to be not progressive or slowly progressive. However, clinical presentations of IRDs can be heterogeneous and sometimes the diagnostic boundaries are not distinct. Although in most cases of IRDs only ophthalmic manifestations are present (nonsyndromic), over 70 forms of syndromic IRDs have been reported (Werdich et al., 2014; OMIM, https://www.ncbi.nlm.nih.gov/omim). The most common one is Usher syndrome (USH), characterized by RP and hearing loss (Tsang et al., 2018). Inheritance can be autosomal recessive (AR), autosomal dominant (AD), or X-linked (XL). Mitochondrial and digenic patterns of inheritance have also been described. To date, over 260 genes have been implicated in the etiology of IRD (RetNet, Retinal Information Network, https://sph.uth.edu/Retnet/). However, the contribution of each gene to the overall prevalence of the disease is relatively small and for many of them only a few reported cases of pathogenic mutations have been reported worldwide. Moreover, in approximately 30% of IRD patients, the underlying genes are still to be discovered. These factors make the genetics of IRDs very challenging (Duncan et al., 2018). To date, many reports have described the clinical and epidemiological characteristics of the various forms of inherited retinal dystrophies in the western world and in countries with specialized hospitals. However, IRD patients coming from regions with limited access to specialized structures to diagnose retinal diseases are strongly understudied or underrepresented in current literature. These patients often receive their first diagnosis of IRD abroad or remain misdiagnosed because of the scarce knowledge in the field of retinal diseases in their countries of origin, thus representing a significant lack of information for the epidemiological and clinical studies worldwide. Here, we report on an ethnically heterogeneous group of IRD patients from different underrepresented countries, referring to Italian Hospitals, describing their clinical and genetic features and expanding the epidemiological spectrum of IRDs in understudied populations.

## Materials and Methods

### Selection of Subjects

The cohort presented in this manuscript was recruited at the IRDs Units of the following Italian hospitals, all members of ERN-EYE: ASST Santi Paolo e Carlo Hospital—University of Milan, Bambino Gesù IRCCS Children’s Hospital—Rome, and Agostino Gemelli hospital/Università Cattolica del Sacro Cuore—Rome. All patients underwent detailed clinical examinations to assess their clinical diagnosis. Ophthalmic examination included best-corrected visual acuity (BCVA), color-vision test, slit-lamp examination, visual field analysis, spectral-domain optical coherence tomography (SD-OCT), fundus autofluorescence (FAF), full-field electroretinogram (ERG), and multifocal electroretinogram (MfERG) recorded according to the ISCEV standards.

All patients received genetic counseling to explain the risks and benefits of genetic testing and gave their informed consent.

The study conformed to the Helsinki declaration of 1984 and subsequent amendments. Ethics Committee approval was obtained (Ethical Committee of Azienda Sanitaria dell’Alto Adige, Italy - Approval No. 132-2020).

### Genetic Testing

Genetic testing was performed at MAGI’s laboratories (MAGI’s Lab s.r.l., Rovereto, TN, Italy, and MAGI Euregio s.c.s., Bolzano, Italy) and at the Genetic Unit of the Bambino Gesù children’s hospital. All protocols and their technical aspects have been published elsewhere (Marceddu et al., 2019; Iarossi et al., 2021).

Briefly, DNA was extracted from blood or saliva using a commercial kit (E.Z.N.A. Blood DNA kit Omega Bio-Tek; Norcross, GA, United States) or from blood with Qiagen columns (QIAamp DNA minikit; Qiagen, Hilden, Germany) according to the manufacturer’s instructions. DNA was analyzed via next-generation sequencing (NGS) on an Illumina MiSeq personal sequencer (Illumina, San Diego, CA) using an in-house bioinformatics pipeline or using the Twist Custom Panel kit (Twist Bioscience, South San Francisco, CA, United States) according to the manufacturer’s protocol on a NovaSeq6000 platform (Illumina, San Diego, CA, United States).

Sanger sequencing, using a Beckman Coulter CEQ 8000 sequencer (Beckmann Coulter, Milan, Italy), was performed to validate all genetic variants identified in the probands and for family segregation studies.

The pathogenicity of all identified variants was evaluated according to American College of Medical Genetics and Genomics guidelines (ACMG) (Richards et al., 2015), with the help of VarSome (https://varsome.com/) (Kopanos et al., 2019), dbSNP (https://www.ncbi.nlm.nih.gov/snp/), ClinVar (https://www.ncbi.nlm.nih.gov/clinvar/) and gnomAD (https://gnomad.broadinstitute.org/) databases. Only variants of unknown significance (VUS), likely pathogenic (LP) variants and pathogenic (P) variants were considered in our analysis and the test was defined as “positive”.

A positive genetic test was considered solved when patients had: pathogenic and/or likely pathogenic variants in dominant and X-linked (XL) genes, even in the absence of family history; VUS variants on dominant and X-linked genes, with the support of family history or of the family segregation study; at least two variants in recessive genes, with a segregation study to verify biallelism. Without a family segregation study, probands carrying ≥2 potentially disease-causing variants were considered unsolved.

### Statistical Analysis

Mann–Whitney test for independent samples was used to compare pairs of medians (MedCalc Sofware, Mariakerke, Belgium). Quantitative data are reported as the median and interquartile range (IQR, QR3-QR1).

### Results

We report the results of genetic analyses on 123 IRD probands, 69 males and 54 females, mean age 41 (IQR, 54–30) years, disease onset 13 (IQR, 27.25–5) years. Pediatric patients (disease onset 0-14 years) represent 52.3% of the whole population. 36.6% of probands had a family history of IRDs, while for 63.4% of probands the family history was absent or unknown and they were defined sporadic cases.

Genetic testing was extended in 28 families, considering 52 probands’ relatives (24 males and 28 females, of which six were affected and 46 were healthy), thus reaching a total of 175 tested individuals. A total of 79 probands (64.2%) carried potentially disease-causing genetic variants, 10 (12.7%) carried variants in AD genes, 61 (77.2%; 30 homozygotes and 31 compound heterozygotes) carried variants in AR genes and 8 (10.1%) carried variants in XL genes.

**FIGURE 1:**
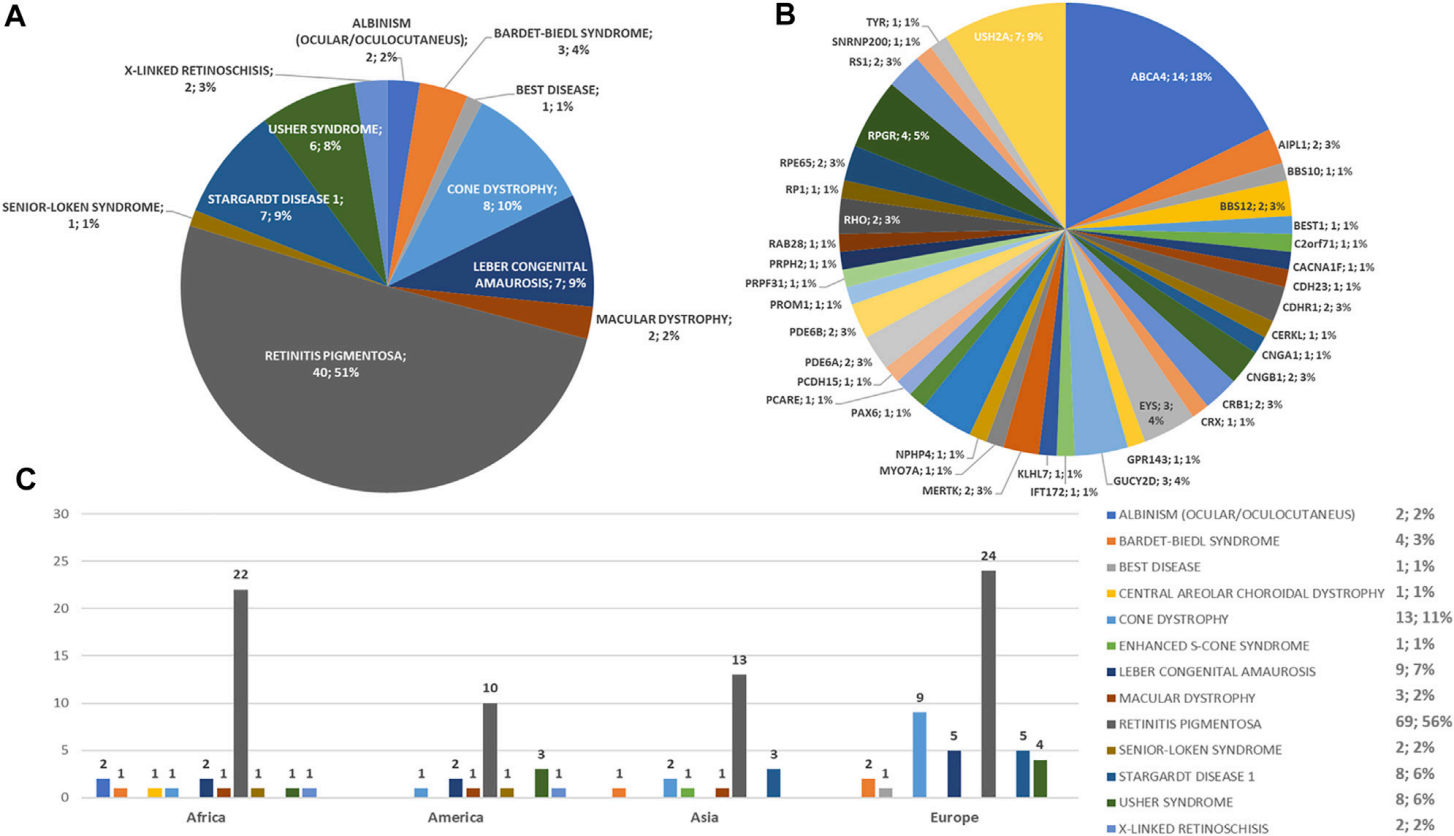
Phenotype and genotype distributions of understudied ethnic groups. Pie charts show **(A)** the phenotypes and **(B)** genotypes of positive cases. **(C)** Bar chart shows the number of examined patients, divided per phenotype.

A summary of the phenotypes tested in the 123 probands and the phenotypes and genes with variants found in the 79 patients who tested positive to the genetic test are reported in Figures 1A–C.

**FIGURE 2:**
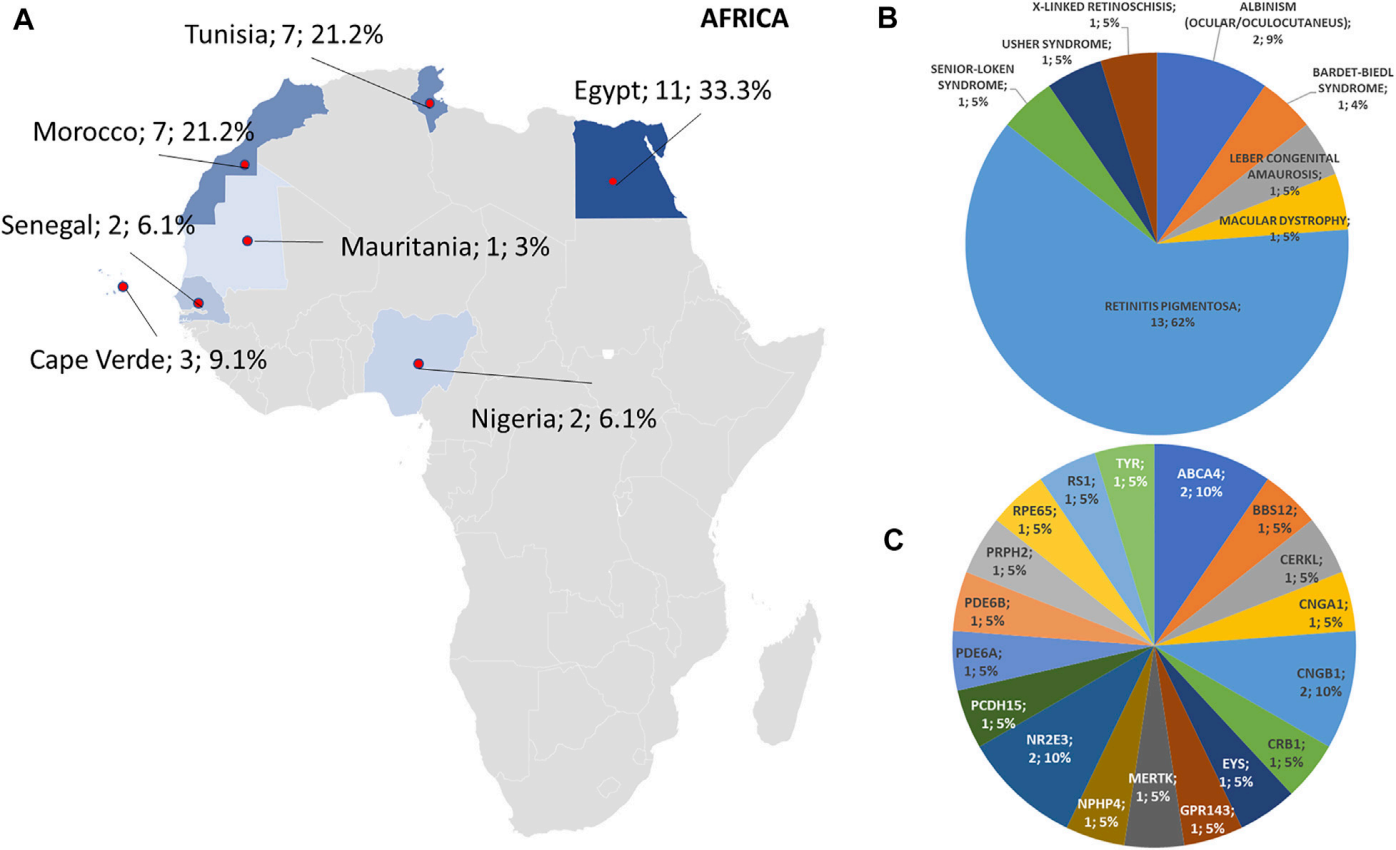
Phenotype and genotype distributions in African countries. **(A)** The map shows the numbers and percentages of examined patients. Pie charts show the **(B)** phenotypes and **(C)** genotypes of positive cases.

**FIGURE 3:**
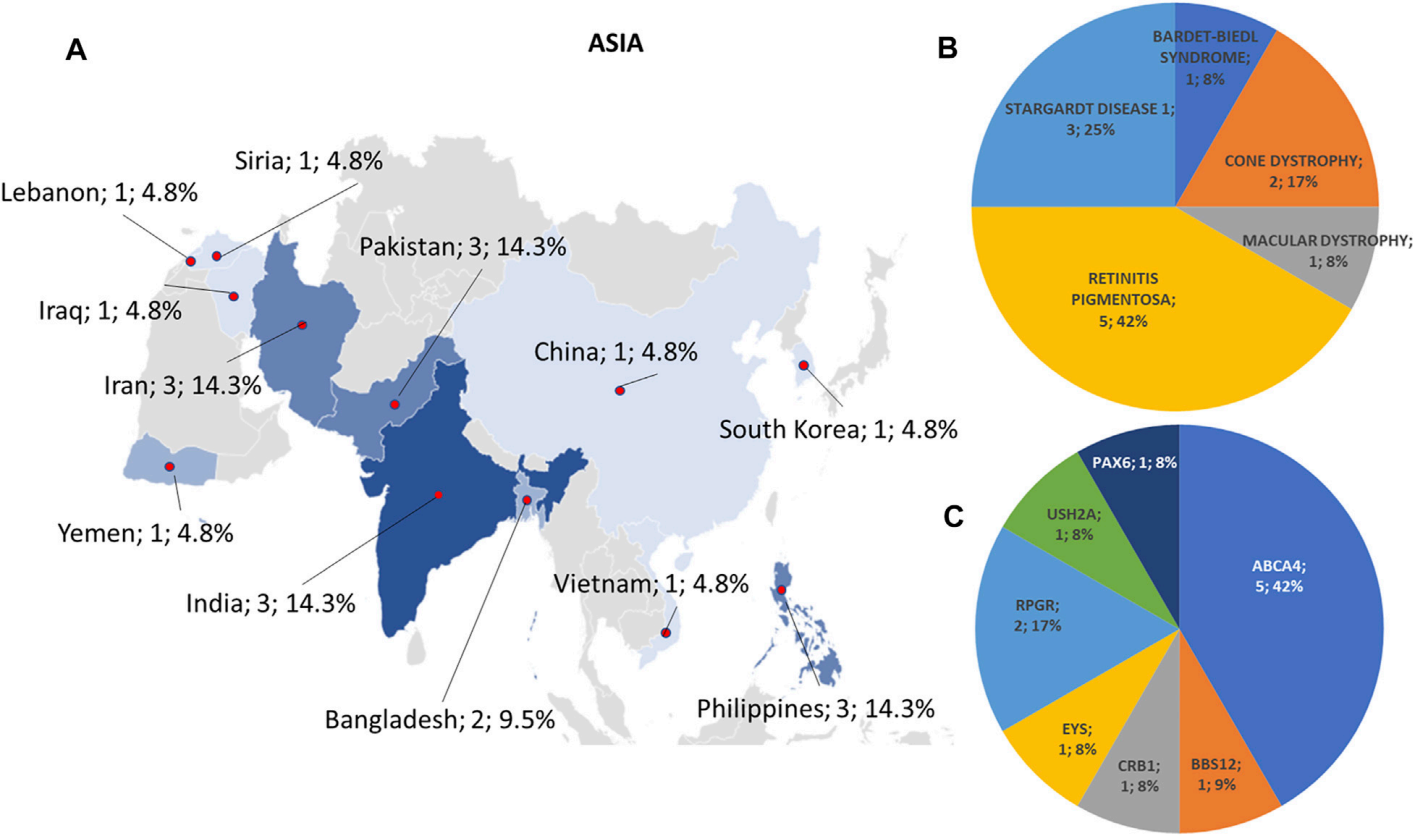
Phenotype and genotype distributions in Asian countries. **(A)** The map shows the numbers and percentages of examined patients. Pie charts show **(B)** the phenotypes and **(C)** genotypes of positive cases.

**FIGURE 4:**
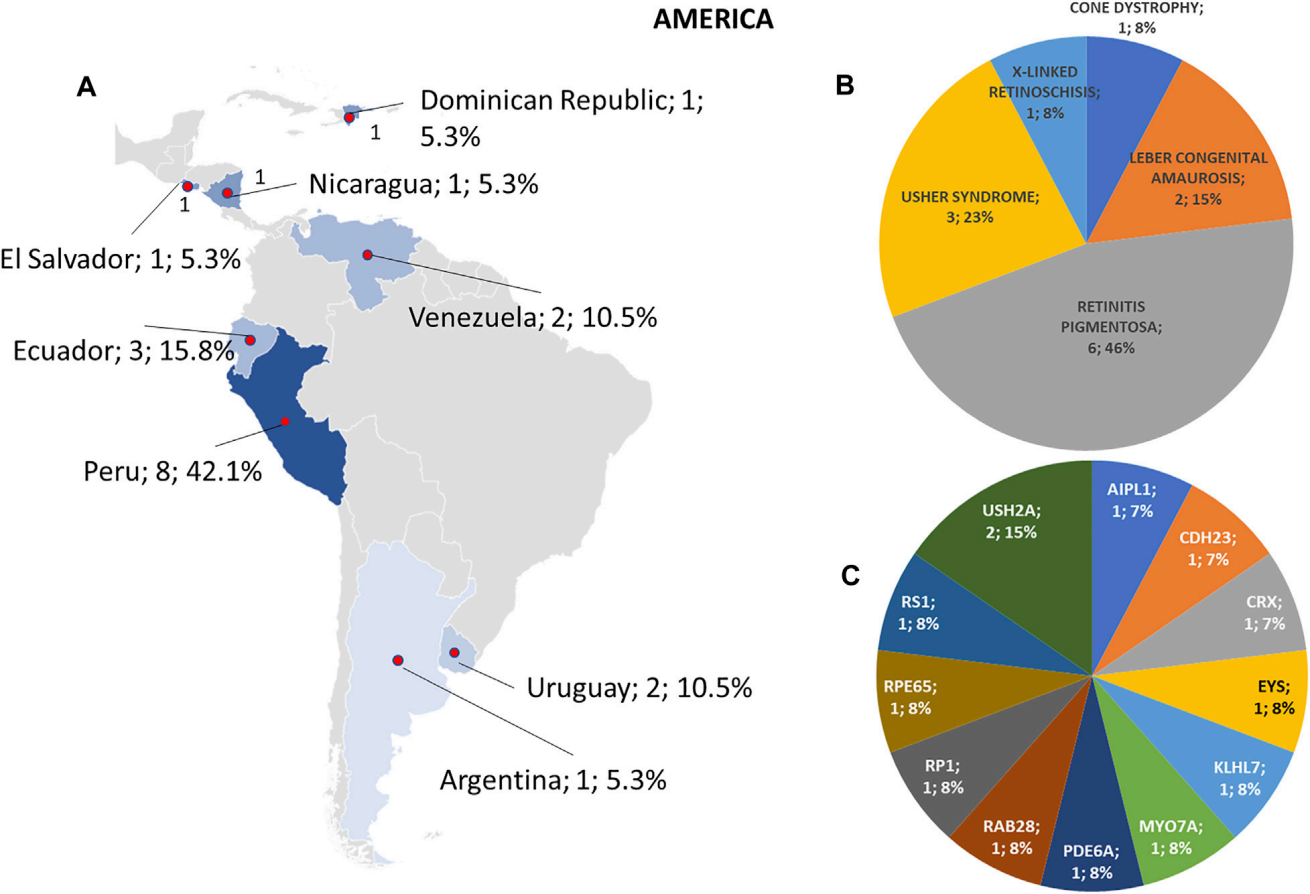
Phenotype and genotype distributions in American countries. **(A)** The map shows the numbers and percentages of examined patients. Pie charts show **(B)** the phenotypes and **(C)** genotypes of positive cases.

**FIGURE 5:**
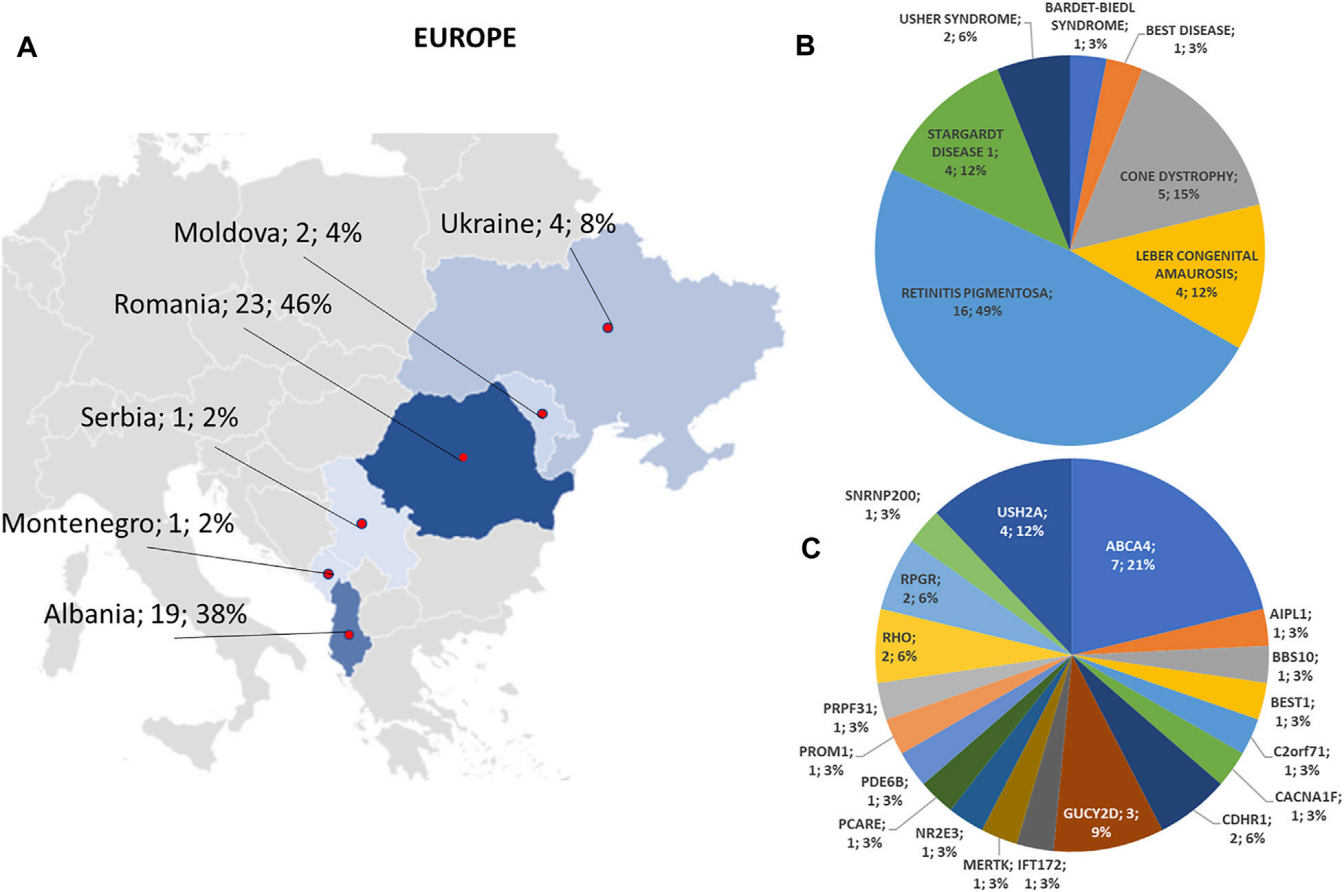
Phenotype and genotype distributions in European countries. **(A)** The map shows the numbers and percentages of examined patients. Pie charts show **(B)** the phenotypes and **(C)** genotypes of positive cases.

Regarding the origins of the patients, 33 out of 123 (26.8%) were Africans (North/Northwest Africa), 21 (17.1%) Asians, 19 (15.4%) Americans (South/Central America) and 50 (40.7%) Europeans (Eastern Europe). The diseases’ distribution across the four continents is shown in Figure 1C. The countries of origin of the probands and the respective distribution of genes and phenotypes are shown in Figures 2–5. Detailed descriptions of probands, phenotypes, and genotypes are listed in Supplementary Table S1-S4. RP was the most represented phenotype (56%), followed by cone dystrophy (11%) and Leber congenital amaurosis (7%), while *ABCA4* was the most frequently mutated gene (18%), followed by *USH2A* (9%) and *RPGR* (5%) (Figure 1). *ABCA4* variants (found in Stargardt disease, macular, and cone dystrophies) were predominant in Asian (42%) and European (21%) probands (Figures 3, 5, respectively). Demographic data, diagnostic yields and inheritance patterns in the different populations are shown in Table 1. The most represented inheritance pattern was the autosomal recessive one for all the four populations, about 50% caused by homozygous variants in all populations except Asians (Table 1).

**TABLE 1:** Characteristics of the studied populations. By comparing the median age at onset of the whole population (13 years, IQR 27.25–5) with those derived from each of the four continents, there was not statistical differences (*p* = 0.29 Africa Vs. All; *p* = 0.26 Asia Vs. All; *p* = 0.89 America Vs. All; *p* = 0.87 Europe Vs. All). Legend: M, male; F, female; AD, autosomal dominant; AR, autosomal recessive; XL, X-linked; HEM, COMP HET, compound heterozygous; HOM, homozygous.

	Probands	M/F	Median age years (QR3-QR1)	Onset years (QR3-QR1)	Positives^a^/Negatives	Families	Affected/Healthy relatives	Sporadics/Familial	AD/AR/XL	AR HOM/COMP HET
**AFRICA**	33/123 (25.4%)	23/10 (67.6%/32.4%)	40 (55.25–33.5)	7 (24.75–1)	21/12 (66.7%/36.4%)	7	2/15 (11.8%/88.2%)	18/15 (54.5%/45.5%)	1/18/2 (9.5%/85.7%/9.5%)	11/6 (64.7%/35.3%)
**ASIA**	21/123 (14.9%)	14/7 (70%/30%)	49 (60–37.5)	16 (31–9)	12/9 (57.1%/42.9%)	2	0/4 (0%/100%)	17/4 (81%/19%)	1/9/2 (8.3%/75%/16.7%)	2/7 (22.2%/77.8%)
**AMERICA**	19/123 (14.2%)	7/12 (36.8%/63.2%)	35 (54–30)	14 (24–8.5)	13/6 (68.4%/31.6%)	7	0/12 (0%/100%)	13/6 (68.4%/31.6%)	3/9/1 (23.1%/69.2%/7.7%)	4/5 (55.6%/44.4%)
**EUROPE**	50/123 (37.3%)	25/25 (50%/50%)	37 (48.5–28)	11.5 (28.5–6.5)	33/17 (66%/34%)	12	4/15 (21.1%/78.9%)	30/20 (60%/40%)	5/25/3 (15.2%/75.8%/9.1%)	13/12 (52%/48%)

a Positive genetic tests comprise both solved and unsolved probands.

A total of 113 different variants were identified (Supplementary Table S5), 33 of which were new (Supplementary Table S6).

## Discussion

**FIGURE 6:**
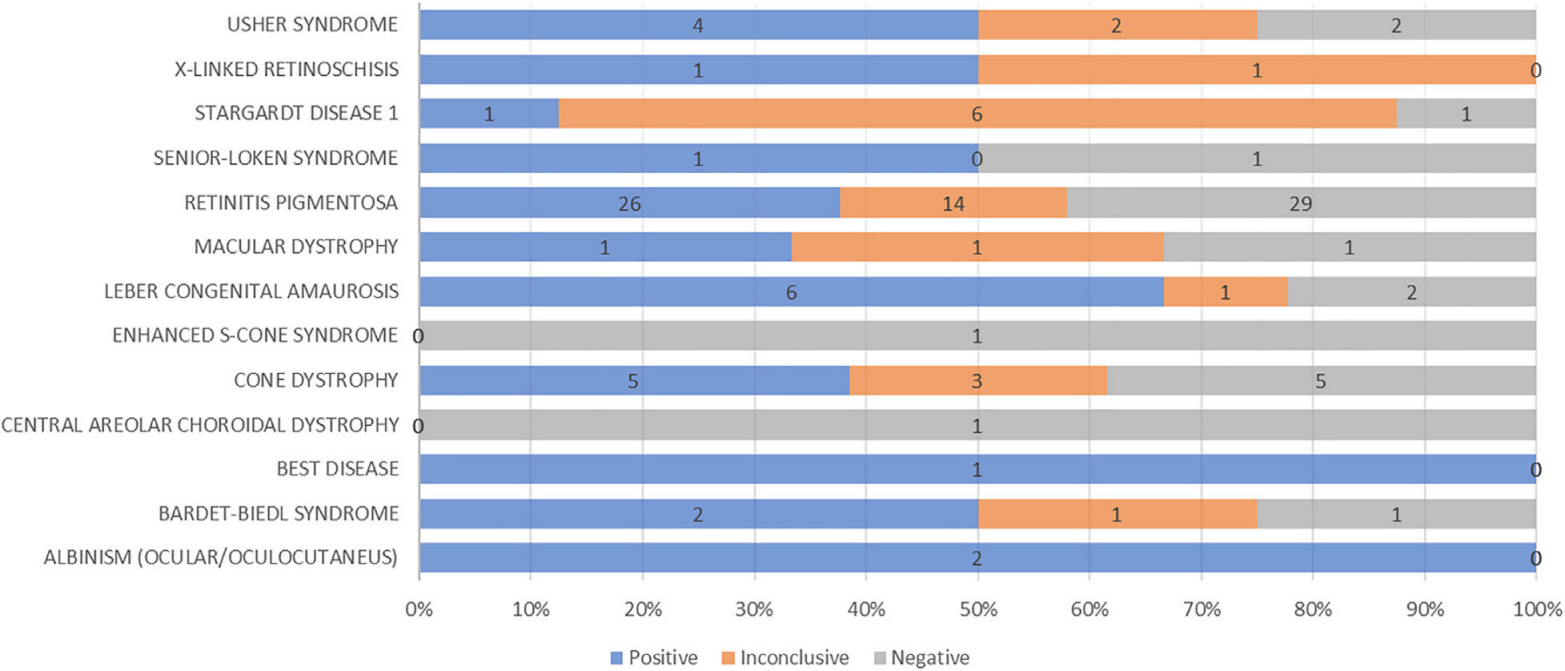
Graphical representation of diagnostic yield for each phenotype analyzed by genetic testing.

Epidemiological and clinical studies on patients affected by IRDs from countries with poorly specialized health systems are largely underrepresented in literature. Only a few reports have been published so far, revealing interesting aspects of the disease manifestations due to the geographic and physical connectivity within families with a higher prevalence of recessive expression caused by consanguinity. Such a situation is particularly advantageous for genetic studies of retinal dystrophies, as it may offer an opportunity to find more prevalent genes in these extended families due to common ancestry. Moreover, the analysis of IRD clinical and genetic expression in understudied populations may improve the epidemiological knowledge of these diseases worldwide. Our previous studies represented the genetics of nonsyndromic RP and USH (Colombo et al., 2021) and macular and cone/cone-rod dystrophy (Falsini et al., 2022) in large Italian series. In this study, we present a similar approach but considering non-Italian patients and families, selected among the understudied ethnic groups referred to Italian specialized hospitals, depicting a picture, though limited in number, of the different expressions of the disease in different world areas. As expected (O'Neal and Luther, 2021), and in line with other reports on understudied ethnic groups, the prevalent disease in our patients is represented by RP, affecting 56% of the patients and reaching a prevalence of 63% with the inclusion Leber congenital amaurosis. Nevertheless, only 37.7% of patients presenting a RP phenotype had a proper genotypic correlation, consistent with the results reported by our group in a cohort of RP and USH patients (Figure 6). As reported in our previous work, also in this case the criteria to deem a case “solved” were very stringent: recessive forms were considered unsolved even if they presented two pathogenic variants but lacked a family segregation study to determine whether the alleles were in *cis*- or *trans*-configuration. Indeed, 50% of the 60 AR patients carried heterozygous variants, but only for 9 out of 30 of them it was possible to confirm the biallelic phase of the variants by family segregation studies (Supplementary Tables S1-S4). Moreover, from a raw analysis of our group of patients across the different regions, RP is more prevalent in patients from Africa than the other continents, whereas cone dystrophy is more represented in patients from Eastern Europe. Among maculopathies, Stargardt disease is the most common one in our group of patients, being more widespread in Asian and Eastern European patients in our cohort.

Although our cohort is not too numerous, it allowed us to make some comparisons with the previously published series regarding the genotypic distribution of IRDs. Considering the whole cohort, our data are in line with the literature: the greatest prevalence is of the *ABCA4* and *USH2A* genes for the AR IRDs, *RHO* and *PRPH2* for the dominant forms and *RPGR* for the x-linked forms (Carrigan et al., 2016; Stone et al., 2017; Hanany et al., 2020; Pontikos et al., 2020; Colombo et al., 2021; Perea-Romero et al., 2021; Falsini et al., 2022). Interestingly, no cases of *USH2A*-related dystrophy were found among Africans, similarly to what was reported by Habibi et al. in a cohort of patients from Tunisia and by Roberts et al. in a small cohort of African patients (Roberts et al., 2016; Habibi et al., 2020). Moreover, no *ABCA4* cases were detected in the South American cohort, in contrast with Motta et al., in which *ABCA4* was the most frequent disease-causing gene among the Brazilian population (Motta et al., 2018).

Comparing the allelic state of the recessive phenotypes, a higher frequency of homozygous patients versus compound heterozygotes emerges (50%/50% in this study; 33.2%/66.8% in nonsyndromic AR-RP and USH; 13.4%/86.6% in AR macular and cone/cone-rod dystrophy) (Colombo et al., 2021; Falsini et al., 2022), reflecting a possible higher frequency of consanguine marriages. This type of marriage is indeed very frequent in the cultures of some of the populations considered here.

This study has some limitations. Although the total number of patients considered is not too small, and the literature associated with the ethnic groups here reported is very scarce, this number is not sufficient to represent realistically the geographic distribution of phenotypes and genotypes and, unfortunately, it does not allow any statistically relevant comparison. Furthermore, in many of the countries reported here, genetic testing is not considered a health priority and the genetics of IRDs remain undetermined, thus limiting the general knowledge on the spread of these diseases. This is why epidemiological studies, such as the one presented here, aim to fill, at least partially, the resulting gaps in this field.

## Data Availability

The original contributions presented in the study are included in the article/Supplementary Material, further inquiries can be directed to the corresponding author.
